# Downregulation of miR-130a, antagonized doxorubicin-induced cardiotoxicity via increasing the *PPAR*γ expression in mESCs-derived cardiac cells

**DOI:** 10.1038/s41419-018-0797-1

**Published:** 2018-07-09

**Authors:** Golnaz Pakravan, Ali Mohammad Foroughmand, Maryam Peymani, Kamran Ghaedi, Motahare-Sadat Hashemi, Mohammadreza Hajjari, Mohammad Hossein Nasr-Esfahani

**Affiliations:** 1 Department of Genetics, Faculty of Science, Shahid Chamran University of Ahvaz, Ahvaz, Iran; 2 Department of Biology, Faculty of Basic Sciences, Shahrekord Branch, Islamic Azad University, Shahrekord, Iran; 3 Department of Biology, Faculty of Sciences, University of Isfahan, Isfahan, Iran; 4 Department of Cellular Biotechnology, Cell Science Research Center, Royan Institute for Biotechnology, ACECR, Isfahan, Iran

## Abstract

Doxorubicin (Dox) is a widely used powerful chemotherapeutic component for cancer treatment. However, its clinical application has been hampered due to doxorubicin-induced cardiomyopathy upon the cessation of chemotherapy. Previous studies revealed that *PPARγ* plays a crucial protective role in cardiomyocytes. Modulation of miRNA expression is an applicable approach for prohibition of toxicity induction. Therefore, the aim of present study is uprising of *PPARγ* transcript levels via manipulation of miRNAs to limit Dox-induced cardiotoxicity in mESCs-derived cardiac cells, as in vitro model cell to provide a simple direct approach for further clinical therapies. Based on bioinformatics data mining, eventually miR-130a was selected to target *PPARγ*. This miRNA is highly expressed in heart. The expression of miR-130a increases sharply upon Dox treatment while specific antagomiR-130a reverses Dox-induced reduced expression of *PPARγ*, cellular apoptosis, and inflammation. Our data strongly suggest that antagomiR-130a limits Dox-induced cellular toxicity via *PPARγ* upregulation and may have clinical relevance to limit in vivo Dox toxicity.

## Introduction

Doxorubicin (Dox) is one of the most commonly used and forceful chemotherapeutic agents in cancer treatment. However, clinical applications of Dox are limited due to its harmful side effects, cumulative and dose-dependent cardiac toxicity and possible risk of cardiomyopathy^1^. Even though the underlying molecular and cellular mechanisms are still unclear, various studies suggest that oxidative stress, calcium overload, mitochondrial damage, cardiomyocyte apoptosis, and autophagy might be involved in Dox toxicity^2^. Nowadays there is an increasing interest to identify new cardioprotective compounds such as propionate derivatives^3^. In this study we have focused on peroxisome proliferator-activated receptors (PPARs) e.g. *PPARγ*. PPARs are ligand-activated transcription factors belonging to nuclear receptor superfamily^4^ that play central roles against cell toxicity and inflammation^5–9^. *PPARγ* not only plays central role in cellular metabolism, it is also a critical player in cardiomyocyte formation and heart development^5^. A previous study has indicated that *PPARγ* agonists inhibited mechanical stress-induced hypertrophy of cultured neonatal rat ventricular cardiomyocytes, through blocking of nuclear factor κB (NF-κB)^10^. Moreover, *PPARγ* activation through a specific agonist caused a cardiomyocytes protection against H_2_O_2_- induced apoptosis via *BCL-2* upregulation, which ultimately could reverse the heart fibrosis in rat^11^. This type of treatment also reduces the size of cardiac infarcts, and enhances the efficiency of cardiac contractility in pig^12^.

MicroRNAs (miRNAs) are key players in gene expression regulation by degradation or destabilization of the target mRNAs^13^. Because miRNAs can affect heart development, function, and disease^14^, their alterations may have therapeutic values or may cause adverse effects to aggravate the pathologic condition. Very recently Zhao et al. have reported that microRNA-140-5p contributes in doxorubicin-induced cardiotoxicity through enhancement of myocardial oxidative stress via targeting NRF2 and SIRT2^2^. On the other hand this group has shown that Dioscin, a natural steroid saponin, alleviates doxorubicin-induced cardiotoxicity via modulation of microRNA-140-5p^15^.

Among enormous miRNAs targeting *PPARγ*, miR-130a might be outstanding due to its heart and lung-restricted expression^16^. MiR-130a first cloned from mouse cerebellum is located at an intergenic region of mouse chromosome 2, with an own promoter (www.mirbase.org). Lee et al. for the first time reported that miR-130a directly targets *PPARγ*^17^. Due to *PPARγ*'s essential role in preventing Dox-induced cardiotoxicity, we examined whether miRNA-130a**-**dependent upregulation of *PPARγ* could reverse the toxicity and apoptosis of mouse embryonic stem cells (mESCs)-derived cardiac cells.

## Results

### Dox-induced cardiotoxicity was testified in mESCs-derived cardiac cells

As previously reported^18^ (Supplementary Fig. 1A), mESCs were shifted to spontaneous cardiac cell differentiation (Supplementary Fig. 1B). Emerging beating embryoid bodies (EBs) were characterized by the expression of cardiac markers, as described in our previous publication^19^ (data not shown). Upon ensuring that adequate amounts of differentiated cardiac cells are yielded, we dissociate beating EBs on day 12 and plate-harvested single cells. Importantly, expression levels of cardiac markers (*α Cardiac actin* and *cardiac α myosin heavy chain* (*α MHC*)) of EBs (day 12) and dissociated cells (day 15) were demonstrated to be similar, ensuing that characteristics of cardiac cells were not altered upon dissociation (Supplementary Fig. 1C). Single cells were then implemented for transfection with anti-miR130a.

Next, we used different Dox concentrations (0.5, 1, 2.5, 5, 10 μM) to acquire effective doses of Dox on cell viability as described in detail in the Materials and Methods section (Supplementary Fig. 2A). We found that 5 μM of Dox suppressed cell viability as much as 50% as assessed by MTS assay (Supplementary Fig. 2B). We also confirmed 5 μM Dox to be effective by using flow cytometry to investigate Annexin V expression level (Supplementary Fig. 2C) and by measuring caspase-3 activity (Supplementary Fig. 2D). Moreover, we observed respectively increased and decreased mRNA levels of *BCL2-associated X protein* (*BAX*) and *B-cell leukemia/lymphoma 2* (*BCL2*) in the treated as compared to the untreated cells verifying apoptotic properties of 5 µM Dox (Supplementary Fig.2E). To our knowledge, cellular responses to Dox-induced inflammation are mediated by *P65*, the functional subunit of NFκβ, which in our experiment was upregulated in treated cells at both RNA and protein levels (Supplementary Fig. 2E to F).

### Dox-induced cardiotoxicity was associated with antiparallel modulation of *PPARγ* with miR-130a expression

**Fig. 1:**
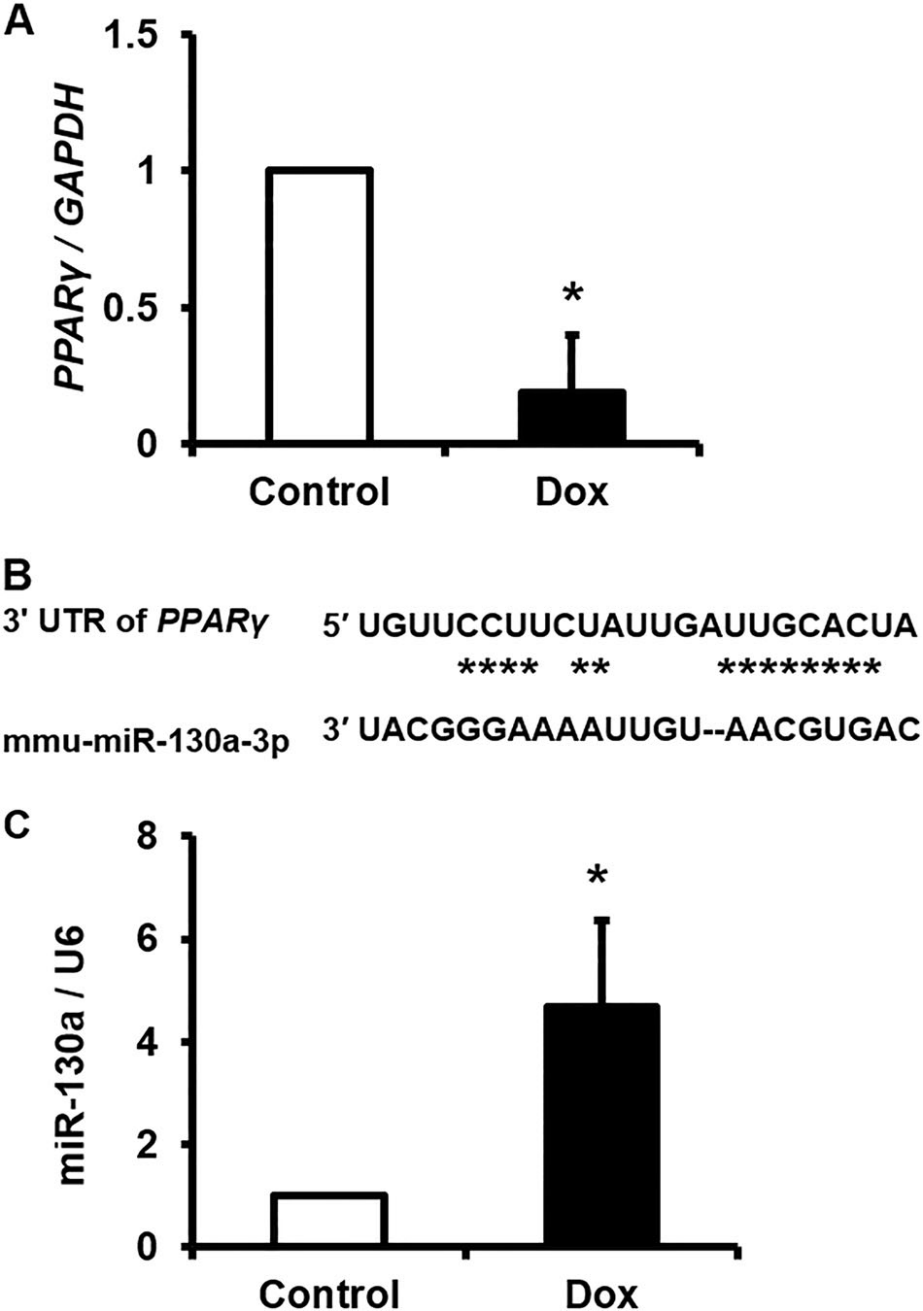
Assessment of *PPARγ* and miR-130a expression upon Dox treatment. **A** Relative expression level of *PPARγ* in Dox-treated cardiac cells (5 µM) compared with the control group (untreated group) as explained in the Materials and Methods section. **B** The seeding region of miR-130a which was deduced from Target Scan. As indicated, miR-130a targets transcripts of *PPARγ* via complementary nucleotides at 3′ UTR. **C** Relative expression level of miR-130a in Dox-treated cardiac cells compared with the control group as explained in the Materials and Methods section. *U6* was chosen as reference gene as indicated. As evident, miR-130a serves as a negative regulator of *PPARγ*. Data are represented as mean ± SEM of three independent replicates of experiment. Star indicates significant difference with control at *p* < 0.05

Upon Dox treatment we observed a strong reduction in *PPARγ* mRNA levels (Fig. 1A). Upregulation of *PPARγ* targeting miRNAs might be responsible for these modulations in *PPARγ* expression. According to our bioinformatics analysis, miR-130a was predicated to target *PPARγ*, specifically in heart tissue (Fig. 1B). Our data indicated upregulation of miR-130a following Dox treatment (Fig. 1C).

### AntimiR-130a transfection enhanced expression of *PPARγ* in control cardiac cells

**Fig. 2:**
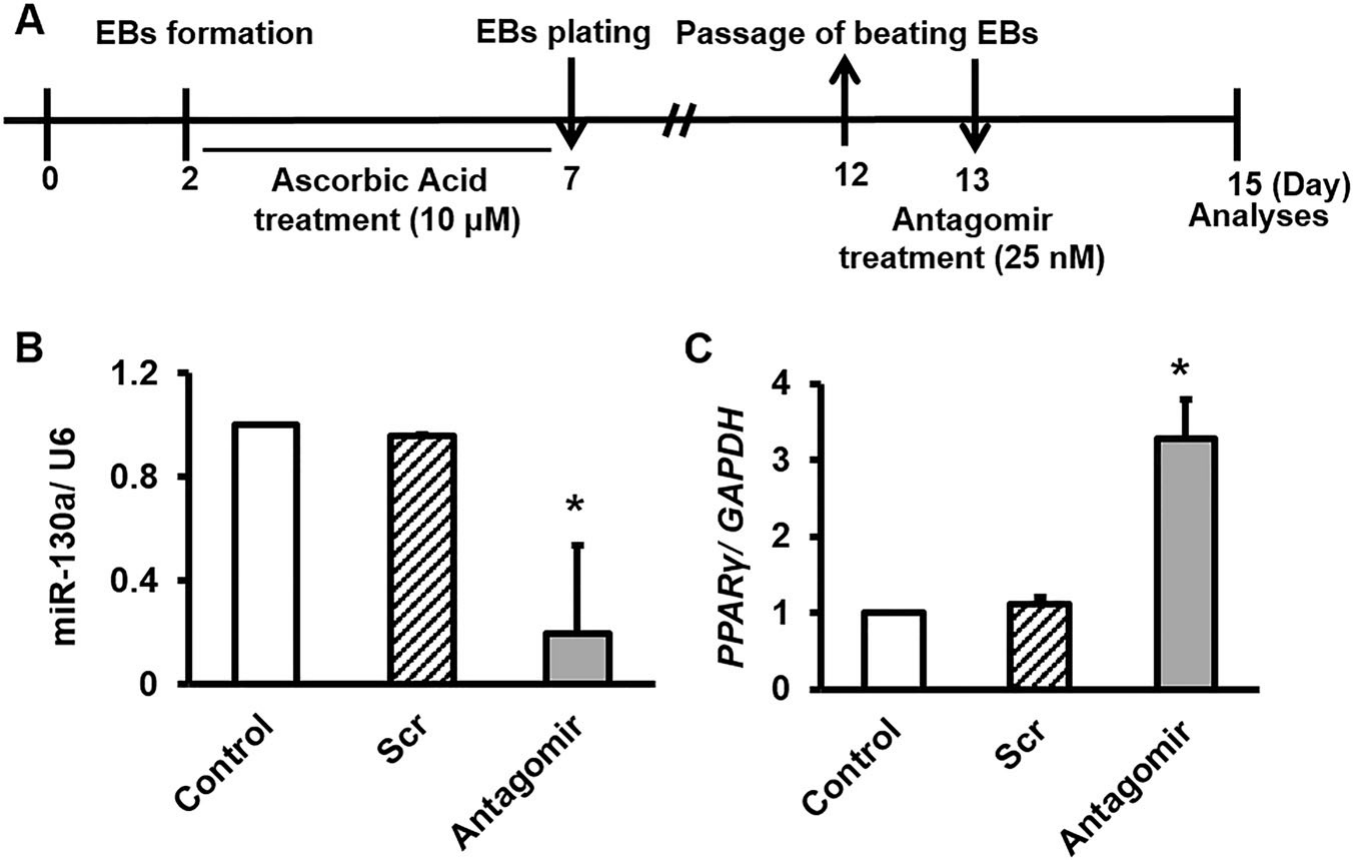
Antagomir-mediated silencing of miR-130a to upregulate expression of *PPARγ*. **A** Schematic representation of protocol used for antagomir transfection. The protocol is similar with the protocol in supplementary Fig. 2A, unless 25 nM of specific antagomir against miR-130a was used. Of note, Dox was not applied to induce stress toxicity. RT-qPCR was performed to assess the relative expression level of miR-130a and *PPARγ*. **B** A decrease in expression level of miR-130a upon antagomir transfection was observed compared with the control samples (Scr: Scramble and control). **C** An increase in *PPARγ* expression was observed post-antagomir transfection compared with the control samples. Data are represented as mean ± SEM of three independent replicates of experiment. Star indicates significant difference with both of scramble and control at *p* < 0.05

To validate *PPARγ*, as bona fide target of miR-130a, we designed an antagomiR for transfection of cardiac cells. As depicted in Fig. 2A, we transfected dissociated cardiac cells with several concentrations (5, 10, 25 nM) of both antimiR-130a and scramble to obtain appropriate amounts of these oligonucleotides for further experiments. Our data indicated that antimiR-130a at concentration of 25 nM not only decreased the miR-130a level, but also significantly enhanced the transcription levels of *PPARγ* (Supplementary Fig. 3A, B). Importantly, similar amounts of scramble (25 nM) was not able to modify miR-130a and *PPARγ* levels. Hereafter, we used 25 nM as the optimal concentration for both oligonucleotides. This concentration was also repeated again in a different set of experiments to confirm reproducibility of our data (Fig. 2B, C).

### AntimiR-130a transfection modulated balanced expression of antiapoptotic markers vs. apoptotic markers without any significant changes in apoptosis in control cardiac cells

We transfected non-Dox-treated cells (Control cells) with antimiR-130a (25 nM). We found that transfection with antimiR-130a (25 nM) did not alter apoptosis in these cells as measured by caspase-3 activity presumably due to inadequate amount of antimiR-130a or inability of this oligonucleotide to suppress apoptotic rate of the control cell (Supplementary Fig. 4A, B). However, the expression level of *BAX* (apoptotic marker) was significantly suppressed contrary to a significant increase in *BCL2* expression (antiapoptotic marker) (Supplementary Fig. 4C). Furthermore, we observed a discrepancy in the expression level of P65 (functional subunit of NFκβ) at RNA and protein levels upon antimiR-130a (25 nM) transfection (Supplementary Fig. 4C, D). We could not explain these observations by our current experiments and further studies are still required. Importantly, we observed downregulation of the functional phosphorylated form of P65 when the cells were transfected with antimiR-130a (Supplementary Fig. 4D), emphasizing the anti-inflammatory properties of antimiR-130a in control cell.

### Pretreatment of antimiR-130a in Dox-treated cells exerted similar trend of *PPARγ* level as control cells

**Fig. 3:**
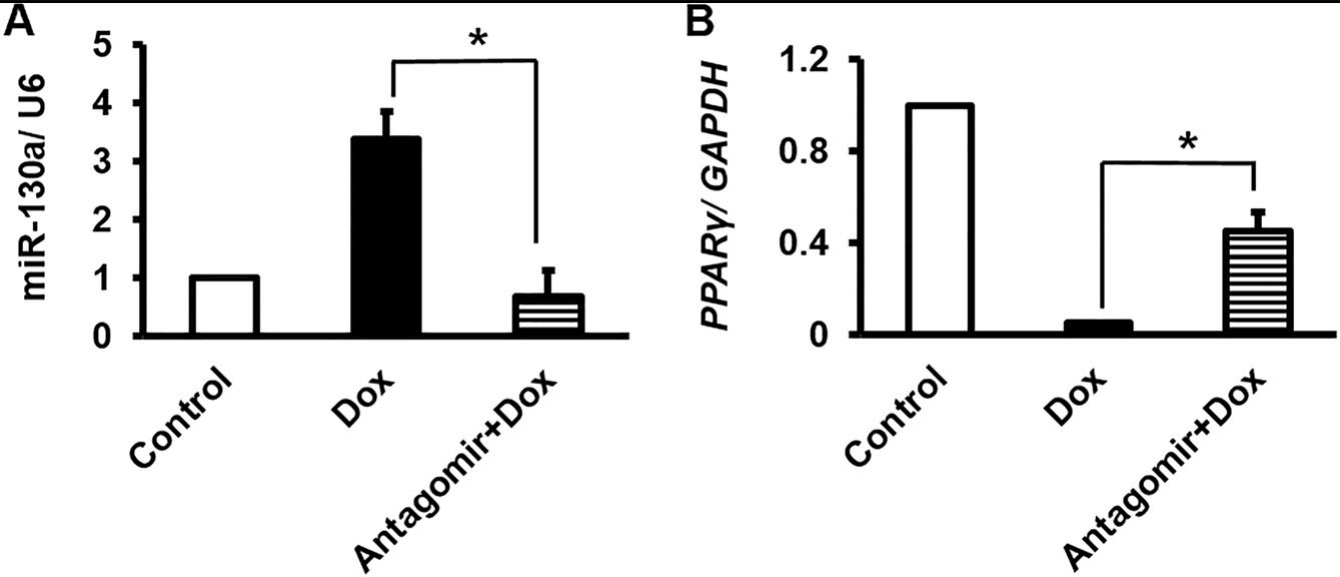
miR-130a-specific antagomir reversed Dox-induced modifications of miR-130a and *PPARγ* transcripts. **A** As obvious, miR-130a-specific antagomir was capable to decrease the expression of miR-130a even after Dox treatment comparable to the control cells. **B** Inversely, transcript level of *PPARγ* was induced by antagomir despite Dox treatment. Star indicates significant difference of antagomir-treated sample with antagomir-untreated counterpart at *p* < 0.05. Reference genes were *GAPDH* and *U6*

To investigate the antimiR-130a effects on cellular Dox-induced toxicity, we transfected cells with antimiR-130a (25 nM) according to the protocol. As expected, miR-130a levels decreased in the Dox-treated group that were pre-transfected with antimiR-130a in an antiparallel trend of *PPARγ* expression (Fig. 3A, B), similar to what we have observed for control cells (Fig. 2B, C).

### AntimiR-130a pretransfection not only modulated balanced expression level of antiapoptotic marker vs. apoptotic marker but also reduced apoptosis rate of Dox-treated cardiac cells

**Fig. 4:**
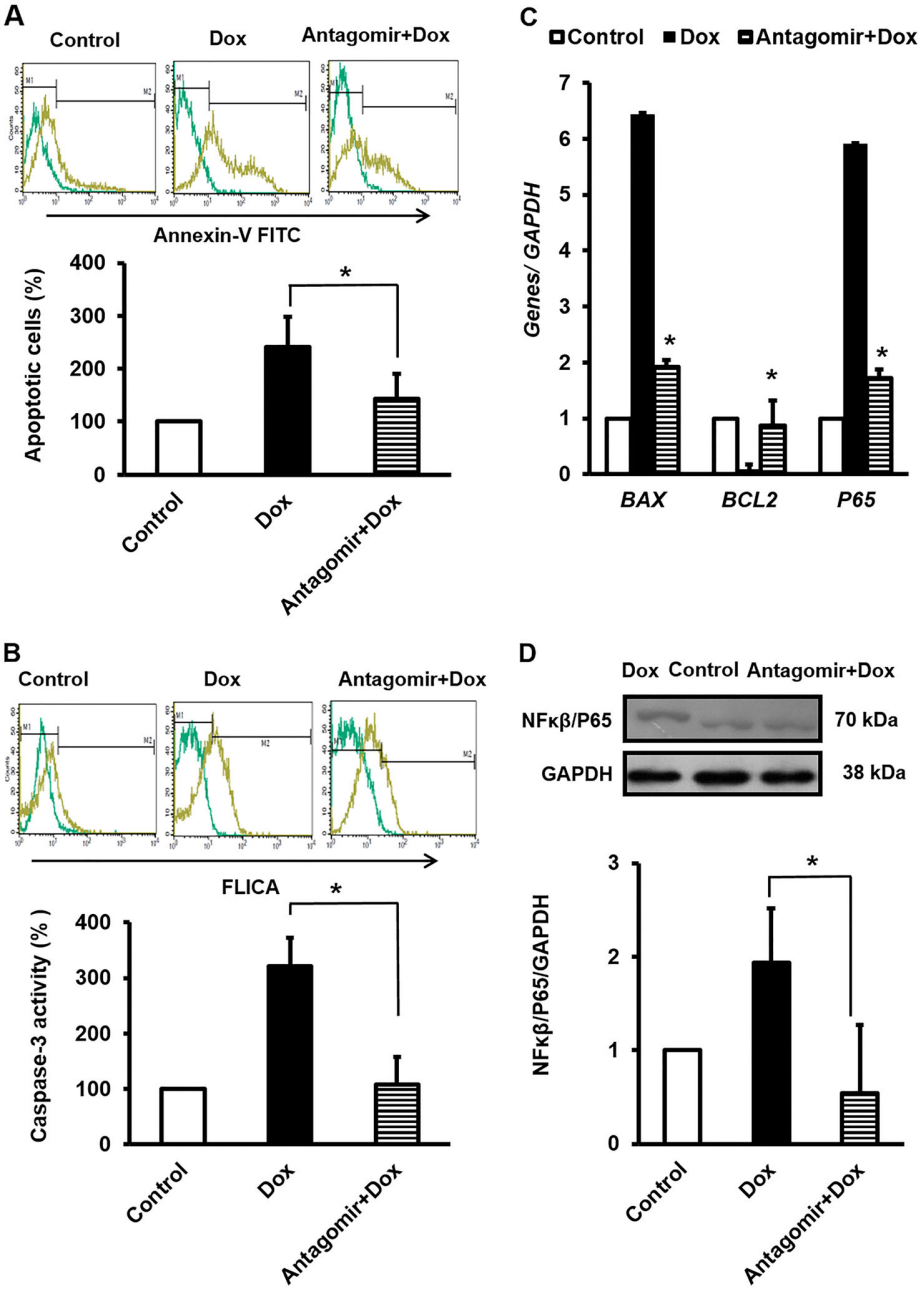
miR-130a-specific antagomir attenuated Dox-induced toxicity. **A** Antagomir reversed Dox toxicity effect as verified by Annexin V-FITC by flow cytometry. **B** In similar condition, caspase-3 activity returned to the baseline level by antagomir pretransfection of cardiac cell prior to Dox induction. **C** RT-qPCR was also performed to assess the relative expression of *BCL-2* and *BAX*, and *NFκB-P65* which clearly represented that miR-130a-specific antagomir had a protective role against Dox. The values are from experiments done in triplicate ± SEM. Reference gene was *GAPDH*. **D** Average relative amount of phosphorylated level of P65, a subunit of NFκB transcription complex to GAPDH in lysate of cardiac cells was reduced after antagomir application. The intensity of each band was quantified by ImageJ software. Remarkably, Dox treatment increased phosphorylated level of P65 thereby, while antagomir prevented this modification. Data are represented as mean ± SEM of three independent replicates of experiment. Star indicates significant difference of antagomir-treated sample with antagomir-untreated counterpart at *p* < 0.05

AntimiR-130a pretransfection effectively downregulated the apoptotic rate of Dox-treated cells, as measured by Annexin V levels and caspase-3 activity (Fig. 4A, B). Suppressed expressions of *BAX* (apoptotic marker) and *P65* vs. increased level of *BCL2* (antiapoptotic marker) were considered as the indicators of decreased apoptosis (Fig. 4C). Moreover, a significant reduction in active form of NFκβ highlighted anti-inflammatory role of antimiR-130a against Dox (Fig. 4D).

### Dox exerted cellular toxicity through miR-130a/*PPARγ* axis

**Fig. 5:**
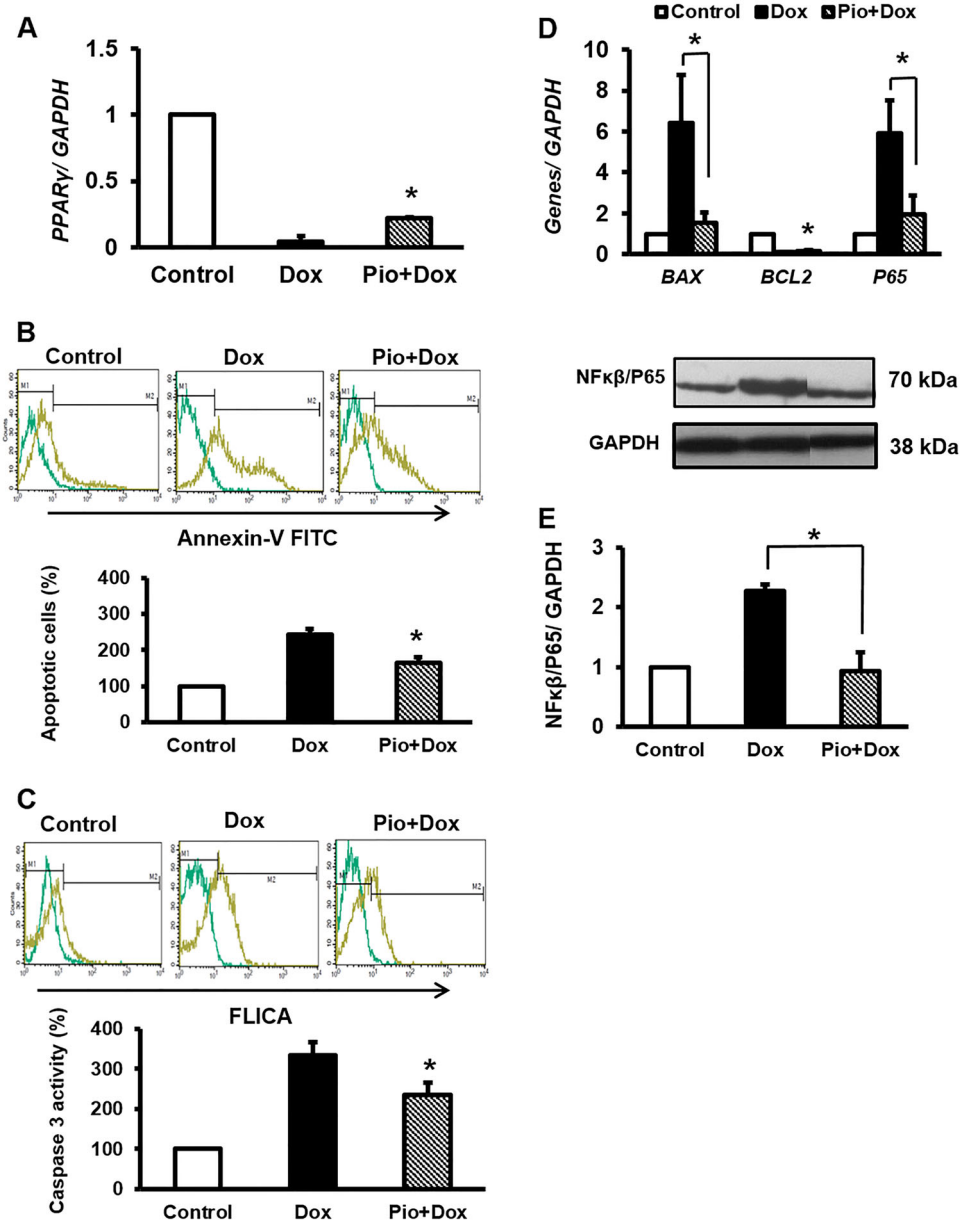
Similar to miR-130a-specific antagomir, Pio pretreatment exerted a protective role against Dox-induced toxicity. Pio was added to the cell culture as described in the Materials and Methods section. **A** Inductive effect of Pio on *PPARγ* expression against attenuating effect of Dox. **B** Pio reversed Dox-induced apoptotic effect as verified by Annexin V-FITC by flow cytometry. **C** Similarly, caspase-3 activity reduced with Pio treatment of cardiac cell prior to Dox induction. **D** Compared with panle (**B**), similar outcomes were deduced on *BCL2* and *BAX* and *NF-κB-P65* expression when cardiac cells were treated with Pio. **E** Relative amount of phosphorylated level of P65 showed a decrease when Pio was used. The intensity of each band was quantified by ImageJ software. The reference gene and protein was GAPDH. Data are represented as mean ± SEM of three independent replicates of experiment. Star indicates significant difference of Pio-treated sample with untreated counterpart at *p* < 0.05

We used effective concentrations^20^ of Pioglitazone (Pio: potent specific PPARγ agonist) prior to Dox treatment in order to confirm whether Dox toxicity is exerted through alterations in both of miR-130a and *PPARγ* expressions, similar to antimiR-130a; we saw that Pio increased *PPARγ* expression, as Pio-activated *PPARγ* had a positive feedback on its expression^21,22^ (Fig. 5A). We also observed that despite Dox implementation apoptosis decreased in Pio-treated cardiac cells (Fig. 5B, C). Therefore, *BAX* and *BCL2* mRNA levels were changed accordingly (Fig. 5D). Comparably to antimiR-130a transfection, P65 was also reduced at both RNA and protein levels (Fig. 5D, E).

### *PPARγ* regulated miR-130a expression reciprocally

**Fig. 6:**
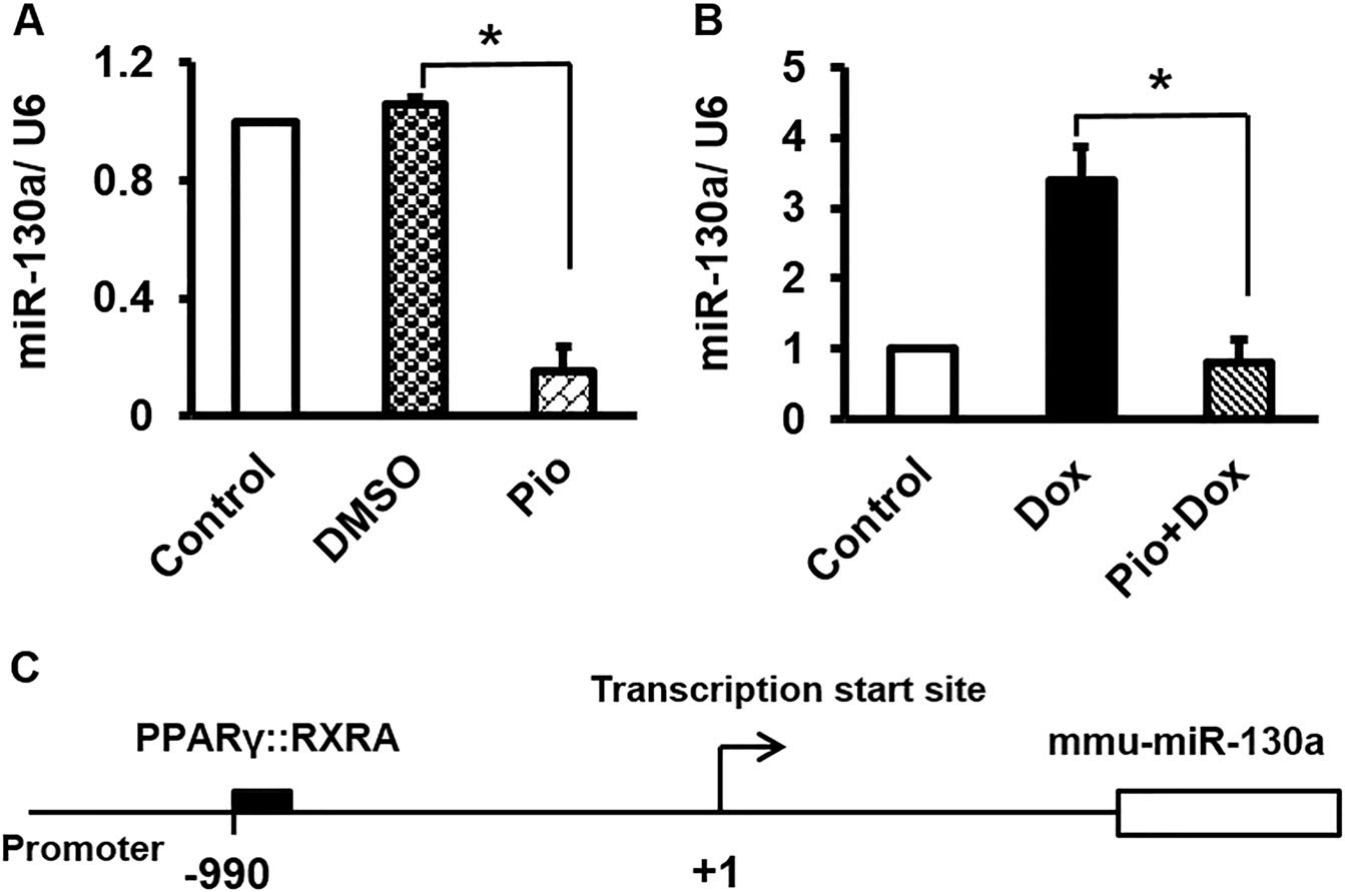
PPARγ may directly attenuate miR-130a through interacting with the respective promoter. **A** Pio treatment exhibited the same effect of miR-130a-specific antagomir on reduction of miR-130a. Incorporation of Pio vehicle (DMSO) on downregulation of miR-130a was excluded as DMSO treatment did not affect the content of miR-130a. **B** Of note, Pio was able to suppress Dox-inductive level of miR-130a. **C** *PPARγ* may directly affect the expression of miR-130a through interacting with its specific response element located at the promoter of miR-130a. The data are deduced from Diana tools, miRGen database (Version 3) (carolina.imis.athena-innovation.gr/diana_tools/web/index)

Surprisingly, we observed a negative feedback for the axis of miR-130a/*PPARγ* as upon Pio treatment miR-130a expression was reduced (Fig. 6A). This outcome was extended to the Dox-treated cells when they were pretreated with Pio (Fig. 6B), demonstrating presence of a feedback by *PPARγ* on miR-130a transcription level. As expected, we found a responsive element in promoter structure of miR-130a for PPARγ:RXRα, when transcription factors binding elements for this promoter were pinpointed trough miRGen database (Fig. 6C). Therefore, it is likely that PPARγ may act as a suppressive factor to attenuate miR-130a expression.

## Discussion

In this study, we delineated that Dox toxicity was exerted via miR-130a that targets *PPARγ*. Antiapoptotic and anti-inflammatory roles of *PPARγ* are well studied^6,23^. Dox, a well-known commercially available antineoplastic drug, serves as a powerful antitumor agent for treatment of human malignancies such as leukemia, lymphoma, breast cancer, and solid tumors^21^. However, a major side effect of Dox that limits its clinical application is its cumulative and dose-dependent cardiac toxicity which may occur immediately upon a single dose (acute effect), after 1 year (chronic effect) or several years post-treatment (delayed effect)^22^. Several different underlying mechanisms are proposed for molecular mechanisms of DOX-induced cardiotoxicity, including cardiomyocyte autophagy, DNA damage, inhibition of topoisomerase II, membrane damage, lipid oxidation, oxidative stress, releases of cardiotoxic cytokines, mitochondrial dysfunction, intracellular calcium overload increased number of lysosomes, activation of extrinsic and intrinsic apoptotic signaling cascades, caspases activation, and inflammatory reactions occurrence^24,25^. Our results are consistent with the previous report by Shalchi Tousi and Sepehri that showed the viability of cardiomyocytes derived from human embryonic stem cell was reduced at 3 and 30 μM doses of Dox^7^. Similarly, several in vivo studies using Dox have shown an increase in caspases-3 activity and NF-κB level, as well as increased expression of *BAX* in left ventricular zone^8,26^. Our data also support previous study by Hosseinzadeh et al. that showed treatment of H9c2 embryonic rat heart-derived cells with Dox, led to an increased *BAX* and caspase-9 expression as well as an enhancement in NF-κβ/P65 level with a significant decrease of *BCL2* expression^27^.

**Fig. 7:**
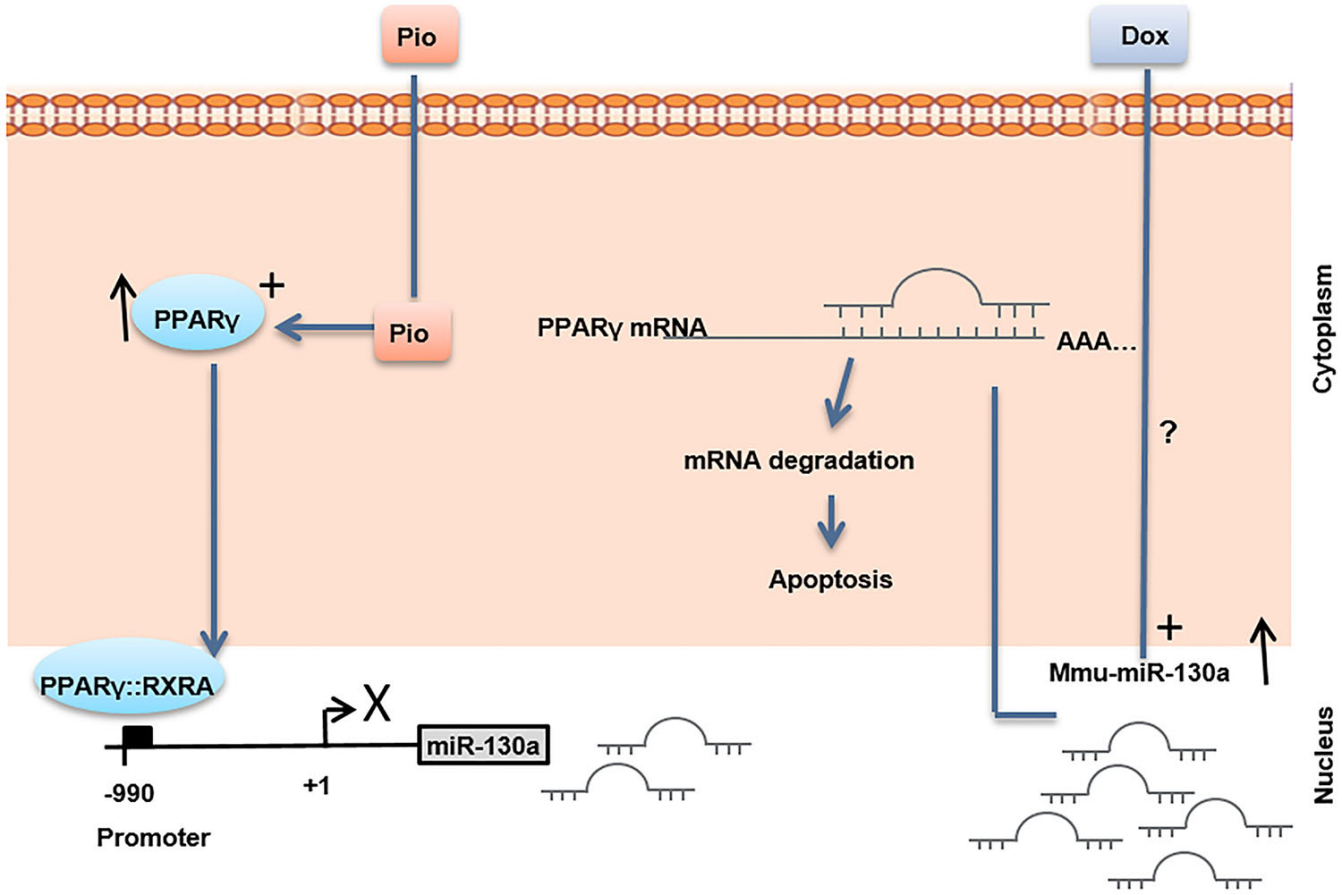
Schematic representation of molecular mechanism of *PPAR*γ-induced attenuation of miR-130a which could serve as potential approach against Dox-induced toxicity of cardiac cells. As depicted Dox-inductive effect on miR-130a increases inflammation leading to apoptosis of cardiac cells, via suppression of cellular *PPAR*γ level. Whereas Pio acts against Dox by increasing amount of activated *PPAR*γ which suppresses miR-130a in cardiac cells

*PPARγ* is one of the key factors for cell resistance to apoptosis and inflammation. Reduction in *PPARγ* expression affecting adipose tissue homeostasis has been reported in white adipose tissue of Dox-treated rats^28,29^. We have also shown that Dox treatment of cardiac cells leads to a significant downregulation of *PPARγ*. Rosiglitazone, a potent synthetic agonist of *PPARγ*, protects rat cardiomyocytes from oxidative stress-induced apoptosis. Similar outcome was obtained upon *PPARγ* overexpression in cardiomyocytes which prevented apoptosis through *BCL2* upregulation^30^. Similarly, *PPARγ* activation has been shown to inhibit the expression of various inflammatory proteins such as iNOS, TNFα, and NF-κβ in several cells types including ventricular cardiomyocytes^31^. Therefore, it is not surprising that *PPARγ* is accounted as a substantial factor for the maintenance and integrity of cardiac cells. In this regard, severe cardiac defects have been documented in *PPARγ* null mice^32^. MiRs are crucial factors in regulating *PPARγ* expression and function. They exert diverse effects on heart function, development, and disease. Among them miR-130a is of interest as it is highly expressed in heart in a tissue-restricted manner^16,33^. Previous report has indicated threefold higher levels of miR-130a in embryonic hearts compared to adult heart, suggesting that miR-130a is a dynamically modulated factor throughout heart development^33^. We have shown that miR-130a expression is upregulated in Dox-treated cardiocytes as an indicator of inflammation. In agreement to our observations, miR-130a has been reported to play a role in metabolism-related inflammation, as its expression was increased in both primary human macrophages and TNFα-treated adipocytes and was declined in murine liver treated with resistin^34^. Furthermore, increased levels of miR-130a have been reported to be correlated with atherosclerosis severity^35^. Upregulation of miR-130a was also reported in patients with multiple cardiac risk factors and stable coronary artery disease and Dahl salt-sensitive rats with heart failure^14,36^. Inhibitory effects of miR-130a on *PPARγ* expression have been already well investigated. Notably, NF-κB/P65 may serve as a positive transcription factor for miR-130a promoter^37^. Therefore, increased expression of miR-130a may enhance own expression assertively via inhibition of *PPARγ* and enhancement of NF-κB/P65 (Fig. 7). Hence, it seems that miR-130a is strategically positioned to enhance own expression manifesting the cardiotoxicity of Dox. To prevent such phenomenon, antisense inhibitor technique is a trustworthy approach. As we elucidated, inhibition of miR-130a was able to reverse Dox toxicity effects through stimulation of *PPARγ* expression. Interestingly, our study showed downregulation of miR-130a by *PPARγ* as a negative feedback. However, it remains unclear and requires further studies to elucidate whether such suppression occurs directly. Taken together, our data indicated a centralized cardio-pathogenic role of miR-130a and highlighted its importance as an approach to control and manage cardiomyopathy progression.

## Materials and methods

### Differentiation of mESCs to beating embryoid bodies

mESC line, Royan B20 (RB20), was used in this study and maintained in undifferentiated state as previously reported^19^. Cells were grown at 37 °C under 5% CO_2_ in adherent culture on 0.1% gelatin-coated tissue culture plates and media were changed at a minimum of every 2 days. For differentiation, approximately 8×10^2^ cells/20 μL of hanging drops were placed in non-adhesive bacterial dishes (Greiner, Germany; 628102) to produce cardiac progenitors as described in our previous publications^18^. Cells were left in tissue culture plates for an additional 5 days to develop EBs including cardiac cells (Supplementary Fig. 1). On day 12, beating EBs were passaged and eventually 6×10^5^ cells were cultured at a rate of 30–50% confluency.

### Viability test assay and determination of the effective concentration of Dox

To evaluate cytotoxicity, nearly 3×10^4^ cardiac cells were seeded in each well of a 96-well tissue culture plate^9^. Dox (Ebedoxo, EBEWE Pharma, Austria) was dissolved in distilled water as vehicle and added to the cells culture at final concentrations of 0.5, 1, 2.5, 5 and 10 μM, 48 h after plating the cell. MTS assay was performed as previously reported.

### Real-time quantitative PCR (RT-qPCR) analysis

RT-qPCR was carried out to determine the gene and miR expression. Total RNA was extracted and cDNA synthesis was achieved as previously reported^20^. RT-qPCR was carried out with ABI PRISM 7500 instrument (Applied Biosystems, Foster, CA, USA) by implementing SYBR Green (TaKaRa). Gene-specific primer pairs were designed by the Beacon designer (Version 7.2, USA) and ordered via Pishgam company (Tehran, Iran) (Supplementary table 1). The primers for miR-130a and U6 were purchased from Parsgenome company (Tehran, Iran). Relative expression level of genes was calculated with the 2^–ΔΔCt^ method using *glyceraldehyde 3-phosphate dehydrogenase* (*GAPDH*) and small nuclear RNA, *U6*, as the control for genes and miR-130a respectively.

### Analysis of apoptosis

The rate of apoptosis was assessed with Annexin V-FITC by flow cytometry as previously reported^38^. To assess the activity of caspase-3, a flow cytometric assay was performed utilizing the CaspaTagTM Caspase-3/7 *In Situ* Assay Kit (Millipore, APT403). Fluorescein was used for detection of active caspase-3 based on fluorochrome inhibitors of caspase (FLICA) as described in the manufacturer’s protocol (Millipore).

### Application of antagomiR-130a

To determine the effective concentration of antagomiR-130a, cells were plated for 24 h. Subsequently, cells were transfected with a range of concentrations 5, 10, 25 nM of either antagomiR-130a (miRCURY LNA™ miR-130a inhibitor, 412568-00, Exiqon) or a scramble sample (199004-00, Exiqon) with lipofectamine 2000 (Invitrogen, USA). Finally, 48 h post-transfection, the expression levels of both miR-130a and *PPARγ* were determined by RT-qPCR. To address the effect of antagomiR-130a on Dox-treated cells, antagomiR-130a (25 nM) was added to the culture cell, 24 h prior to Dox treatment (5 µM). Moreover, cells were harvested 24 h after treatment with Dox.

### Application of Pio

Pio (10 µM) was added to the cell medium, 24 h prior to Dox treatment. After medium change, again Pio was added to the cell culture simultaneously with Dox addition.

### Western blotting

Immunoblotting of lysate cells was carried out accordingly^38^. Very briefly, PVDF membranes (PVDF; Biorad, Hercules, CA, USA) were incubated with primary antibodies (1:5000 dilution of mouse anti-GAPDH (Sigma-Aldrich, A2228)) and rabbit anti-NF-κB/P65 antibody (Abcam, ab90532, dilution: 1/500) that specifically recognizes phosphorylated NF-κB for 90 min. Subsequent to washing, membranes were incubated again with horseradish peroxidase (HRP)-conjugated goat antibody against mouse IgG (BBI, Boston Biomedical Inc., Cambridge, MA, USA) at a dilution of 1:16,000, or HRP-conjugated goat anti-rabbit IgG (Sigma-Aldrich) at a dilution of 1:5000 for 45 min.

### Bioinformatics studies

First, to list the putative *PPARγ-*targeted miRNAs, a vast search in mirWalk database (http://zmf.umm.uni-heidelberg.de/apps/zmf/mirwalk2/) was performed. To narrow down the candidate miRNAs, high score miRNAs were selected for data mining in PubMed (https://www.ncbi.nlm.nih.gov/pubmed/). Eventually miR-130a was selected as the best validated heart-specific miR to repress the expression of *PPARγ*. Subsequently characterization of miR-130a was carried out by looking for at several databases such as TargetScan 5.2 (http://www.targetscan.org/mmu_71/), miRanda (http://34.236.212.39/microrna/home.do), miRBase (http://www.mirbase.org/), and miRTarBase (http://mirtarbase.mbc.nctu.edu.tw/php/index.php). In order to characterize miR-130a promoter and its regulators, DIANA-miRGen v3.0 (http://carolina.imis.athena-innovation.gr/diana_tools/web/index) was used^19^.

### Statistical analysis

Tukey’s post-hoc test with one-way analysis of variance and independent Student’s *t*-test were used to identify statistical differences between groups. All experiments were replicated at least in three independent experiments. Values are represented as mean ± SEM (standard error of mean).

## Electronic supplementary material

Supplementary Figure 1

Supplementary Figure 2

Supplementary Figure 3

Supplementary Figure 4

Supplementary Figure 5

Supplementary figure legends

Supplementary table

## References

[1] Minotti G, Menna P, Salvatorelli E, Cairo G, Gianni L (2004). Anthracyclines: molecular advances and pharmacologic developments in antitumor activity and cardiotoxicity. Pharmacol. Rev..

[2] Zhao L (2017). MicroRNA-140-5p aggravates doxorubicin-induced cardiotoxicity by promoting myocardial oxidative stress via targeting Nrf2 and Sirt2. Redox Biol..

[3] Danilenko, L. M. Doxorubicin-associated cardiomyopathy: new approaches to pharmacological correction using 3-(2, 2, 2-trimethylhydrazinium) propionate derivatives. *Res Results Pharmacol.* **4**, 1319–1323 (2018).

[4] Desvergne B, Wahli W (1999). Peroxisome proliferator-activated receptors: nuclear control of metabolism. Endocr. Rev..

[5] Huss JM, Kelly DP (2004). Nuclear receptor signaling and cardiac energetics. Circ. Res..

[6] Fong WH, Tsai HD, Chen YC, Wu JS, Lin TN (2010). Anti-apoptotic actions of ppar-γ against ischemic stroke. Mol. Neurobiol..

[7] Tousi, M. S. & Sepehri, H. The effect of doxorubicin on viability and morphology of human embryonic stem cell-derived cardiomyocytes. *J. Chem. Health Risks* **4**, 1–6 (2014).

[8] Ueno M (2006). Doxorubicin induces apoptosis by activation of caspase-3 in cultured cardiomyocytes in vitro and rat cardiac ventricles in vivo. J. Pharmacol. Sci..

[9] Dehghani L (2013). The influence of dexamethasone administration on the protection against doxorubicin-induced cardiotoxicity in purified embryonic stem cell-derived cardiomyocytes. Tissue Cell.

[10] Yamamoto K, Ohki R, Lee RT, Ikeda U, Shimada K (2001). Peroxisome proliferator-activated receptor γ activators inhibit cardiac hypertrophy in cardiac myocytes. Circulation.

[11] da Silva Torres T, Aguila MB, Mandarim-de-Lacerda CA (2010). Rosiglitazone reverses cardiac adverse remodeling (fibrosis and vascularization) in perinatal low protein rat offspring. Pathol.-Res. Pract..

[12] Palee S, Weerateerangkul P, Surinkeaw S, Chattipakorn S, Chattipakorn N (2011). Effect of rosiglitazone on cardiac electrophysiology, infarct size and mitochondrial function in ischaemia and reperfusion of swine and rat heart. Exp. Physiol..

[13] Bartel DP (2004). MicroRNAs: genomics, biogenesis, mechanism, and function. Cell.

[14] Vickers KC, Rye KA, Tabet F (2014). MicroRNAs in the onset and development of cardiovascular disease. Clin. Sci..

[15] Zhao L (2018). Protective effect of dioscin against doxorubicin-induced cardiotoxicity via adjusting microRNA-140-5p-mediated myocardial oxidative stress. Redox Biol..

[16] Lagos-Quintana M (2002). Identification of tissue-specific microRNAs from mouse. Curr. Biol..

[17] Lee EK (2011). miR-130 suppresses adipogenesis by inhibiting peroxisome proliferator-activated receptor γ expression. Mol. Cell. Biol..

[18] Peymani M, Ghaedi K, Irani S, Nasr-Esfahani MH (2016). Peroxisome proliferator-activated receptor γ activity is required for appropriate cardiomyocyte differentiation. Cell J..

[19] Nazem S (2017). Fndc5 knockdown induced suppression of mitochondrial integrity and significantly decreased cardiac differentiation of mouse embryonic stem cells. J. Cell. Biochem..

[20] Peymani, M. et al. Ameliorating the effect of pioglitazone on LPS-induced inflammation of human oligodendrocyte progenitor cells. *Cell. Mol. Neurobiol*. **38**, 1−11 (2017). 10.1007/s10571-017-0500-6 PMC11481965 28488008

[21] Zafar, M. Z. & Sabir, H. Pharmacological study and overcome the cardiotoxicity associated with anticancer drug doxorubicin. *Qual. Prim. Care* **25**, 368–371 (2017).

[22] Alexieva B (2014). Insights into mechanisms of doxorubicin cardiotoxicity. J. Physiol. Pharmacol. Adv..

[23] Szanto A, Nagy L (2008). The many faces of PPARγ: anti-inflammatory by any means?. Immunobiology.

[24] Renu K, Abilash V, Pichiah PT, Arunachalam S (2017). Molecular mechanism of doxorubicin-induced cardiomyopathy—an update. Eur. J. Pharmacol..

[25] Mobaraki M (2017). Molecular mechanisms of cardiotoxicity: a review on major side-effect of doxorubicin. Indian J. Pharm. Sci..

[26] Abdel‐Raheem IT, Taye A, Abouzied MM (2013). Cardioprotective effects of nicorandil, a mitochondrial potassium channel opener against doxorubicin‐induced cardiotoxicity in rats. Basic Clin. Pharmacol. Toxicol..

[27] Hosseinzadeh L (2011). Curcumin potentiates doxorubicin-induced apoptosis in H9c2 cardiac muscle cells through generation of reactive oxygen species. Food Chem. Toxicol..

[28] Biondo LA (2016). Impact of doxorubicin treatment on the physiological functions of white adipose tissue. PLoS ONE.

[29] Arunachalam S, Tirupathi Pichiah P, Achiraman S (2013). Doxorubicin treatment inhibits PPARγ and may induce lipotoxicity by mimicking a type 2 diabetes‐like condition in rodent models. FEBS Lett..

[30] Ren Y (2009). PPAR gamma protects cardiomyocytes against oxidative stress and apoptosis via Bcl-2 upregulation. Vasc. Pharmacol..

[31] Lehrke M, Lazar MA (2005). The many faces of PPARγ. Cell.

[32] Barak Y (1999). PPARγ is required for placental, cardiac, and adipose tissue development. Mol. Cell.

[33] Kim GH, Samant SA, Earley JU, Svensson EC (2009). Translational control of FOG-2 expression in cardiomyocytes by microRNA-130a. PLoS ONE.

[34] Zheng H (2016). Regulation and mechanism of mouse miR-130a/b in metabolism-related inflammation. Int. J. Biochem. Cell Biol..

[35] Li T (2011). Identification of miR-130a, miR-27b and miR-210 as serum biomarkers for atherosclerosis obliterans. Clin. Chim. Acta.

[36] Dickinson BA (2013). Plasma microRNAs serve as biomarkers of therapeutic efficacy and disease progression in hypertension‐induced heart failure. Eur. J. Heart Fail..

[37] Huang JY (2015). MicroRNA-130a can inhibit hepatitis B virus replication via targeting PGC1α and PPARγ. RNA.

[38] Safaeinejad Z (2017). Resveratrol promotes human embryonic stem cells self-renewal by targeting SIRT1-ERK signaling pathway. Eur. J. Cell Biol..

