# Supplementary figures and images for: Downregulation of miR-130a, antagonized doxorubicin-induced cardiotoxicity via increasing the PPARγ expression in mESCs-derived cardiac cells
Source: Cell Death Dis. 2018 Jul 9;9(7):758. doi: 10.1038/s41419-018-0797-1 (PMC6037713; doi:10.1038/s41419-018-0797-1)

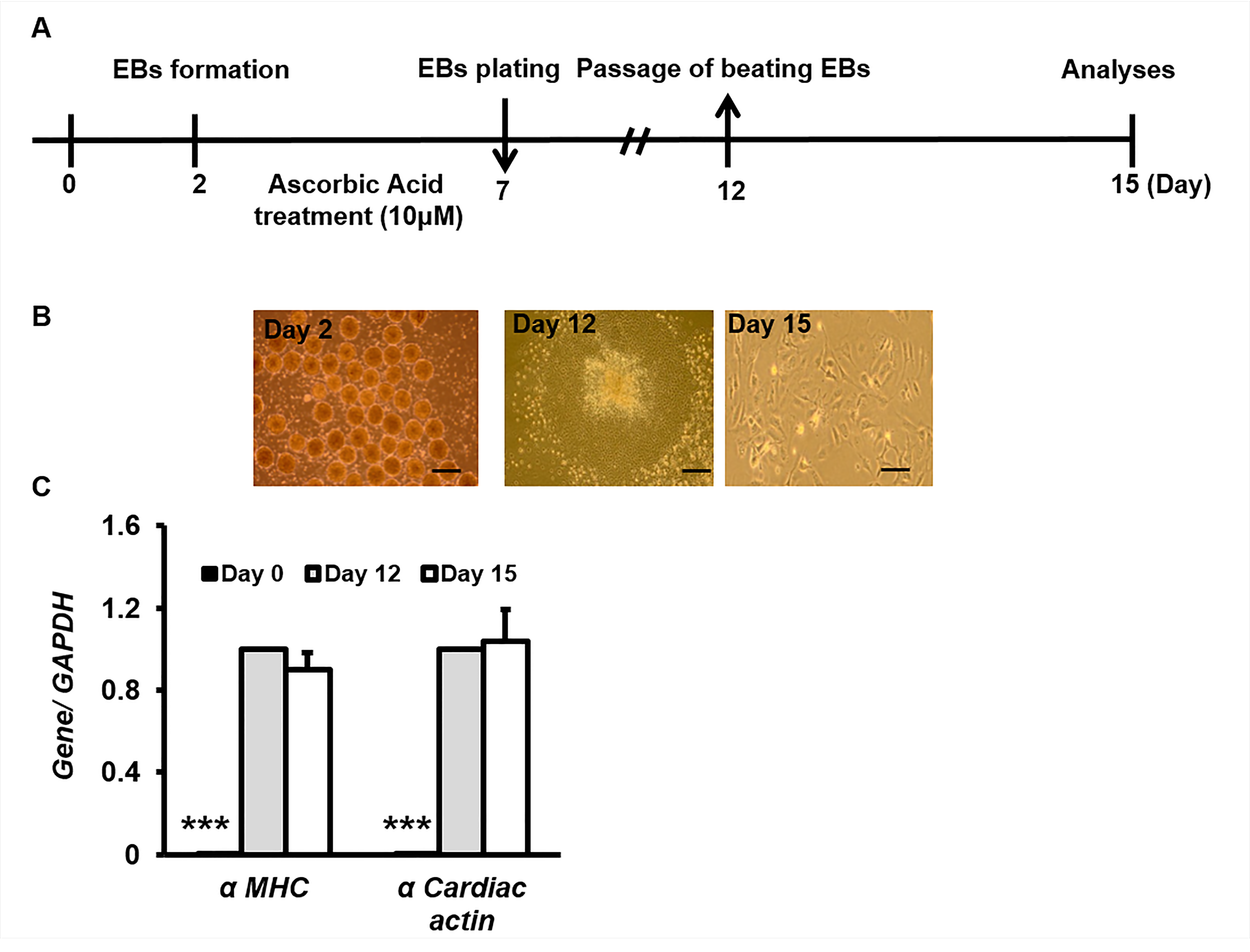

Supplement: Supplementary file 1 — Supplementary Figure 1 [file 41419_2018_797_MOESM1_ESM.tif]

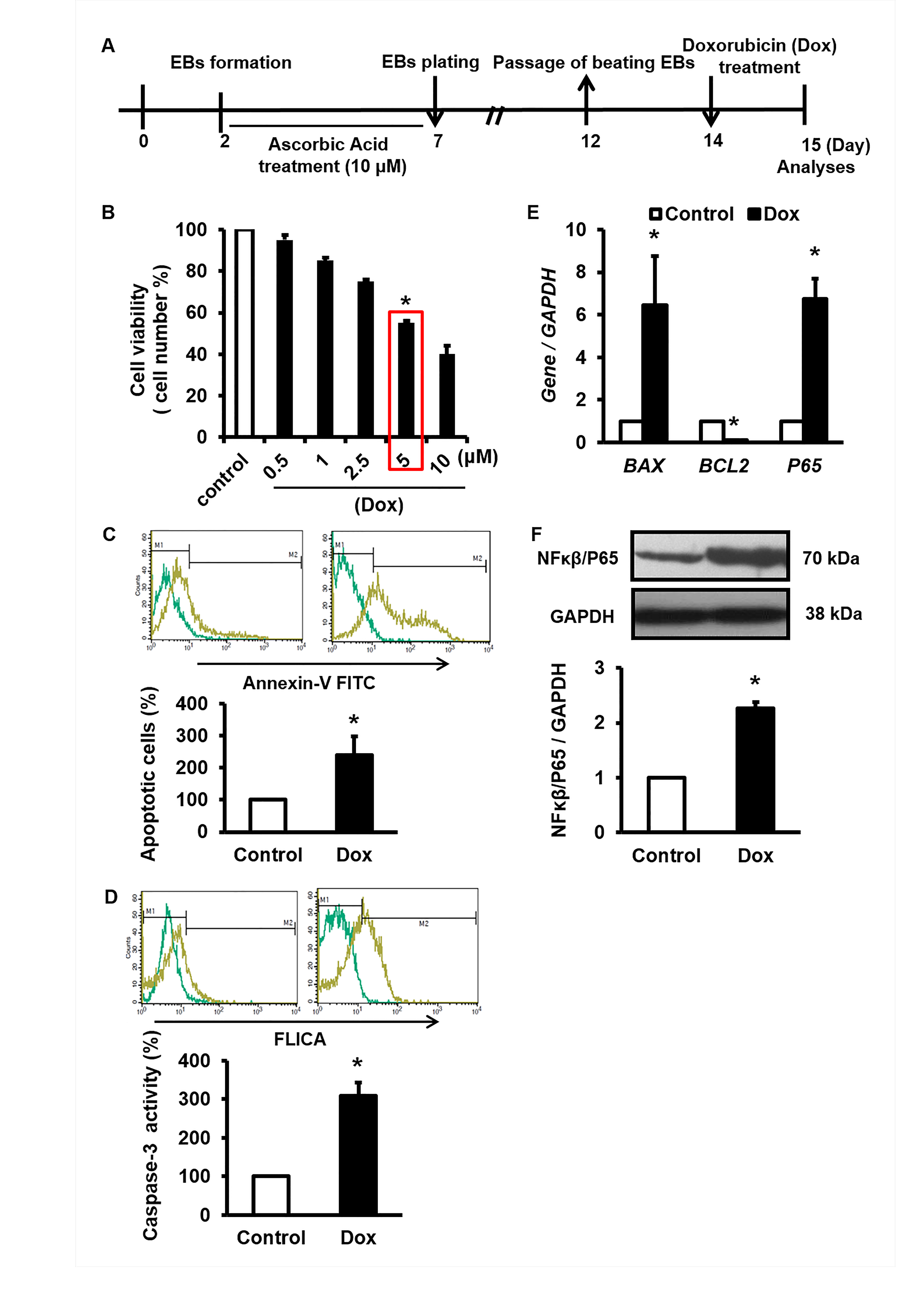

Supplement: Supplementary file 2 — Supplementary Figure 2 [file 41419_2018_797_MOESM2_ESM.tif]

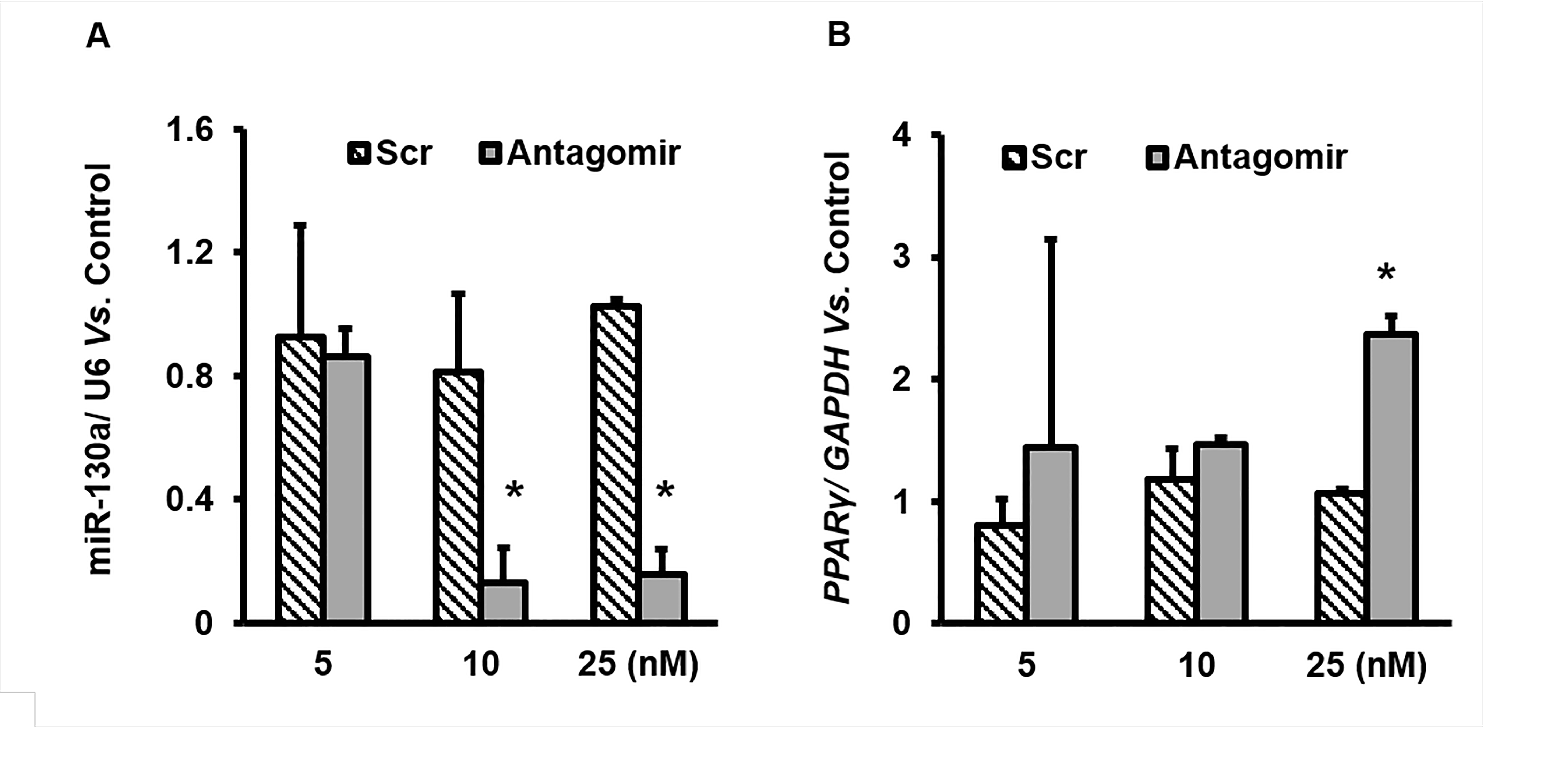

Supplement: Supplementary file 3 — Supplementary Figure 3 [file 41419_2018_797_MOESM3_ESM.tif]

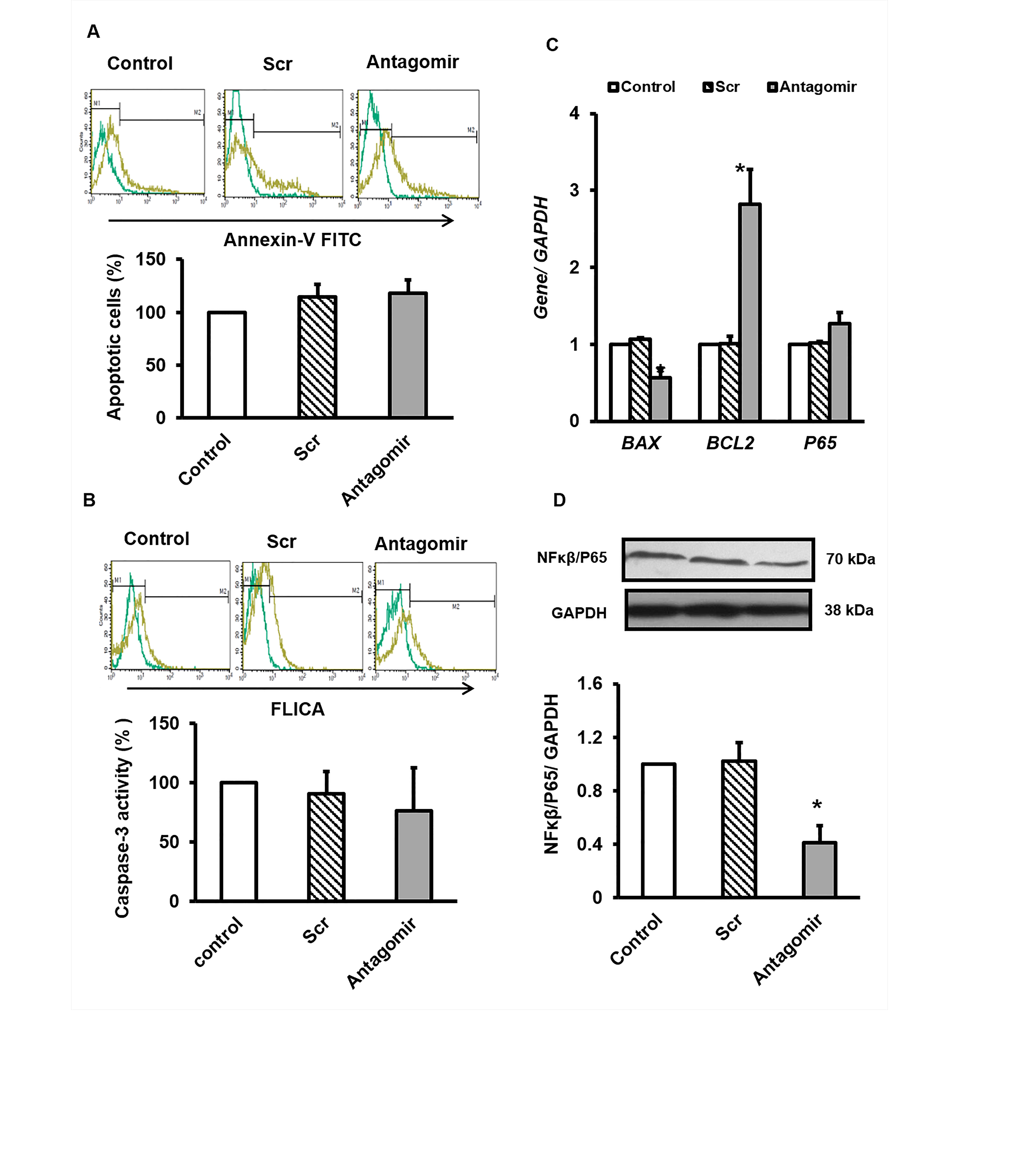

Supplement: Supplementary file 4 — Supplementary Figure 4 [file 41419_2018_797_MOESM4_ESM.tif]

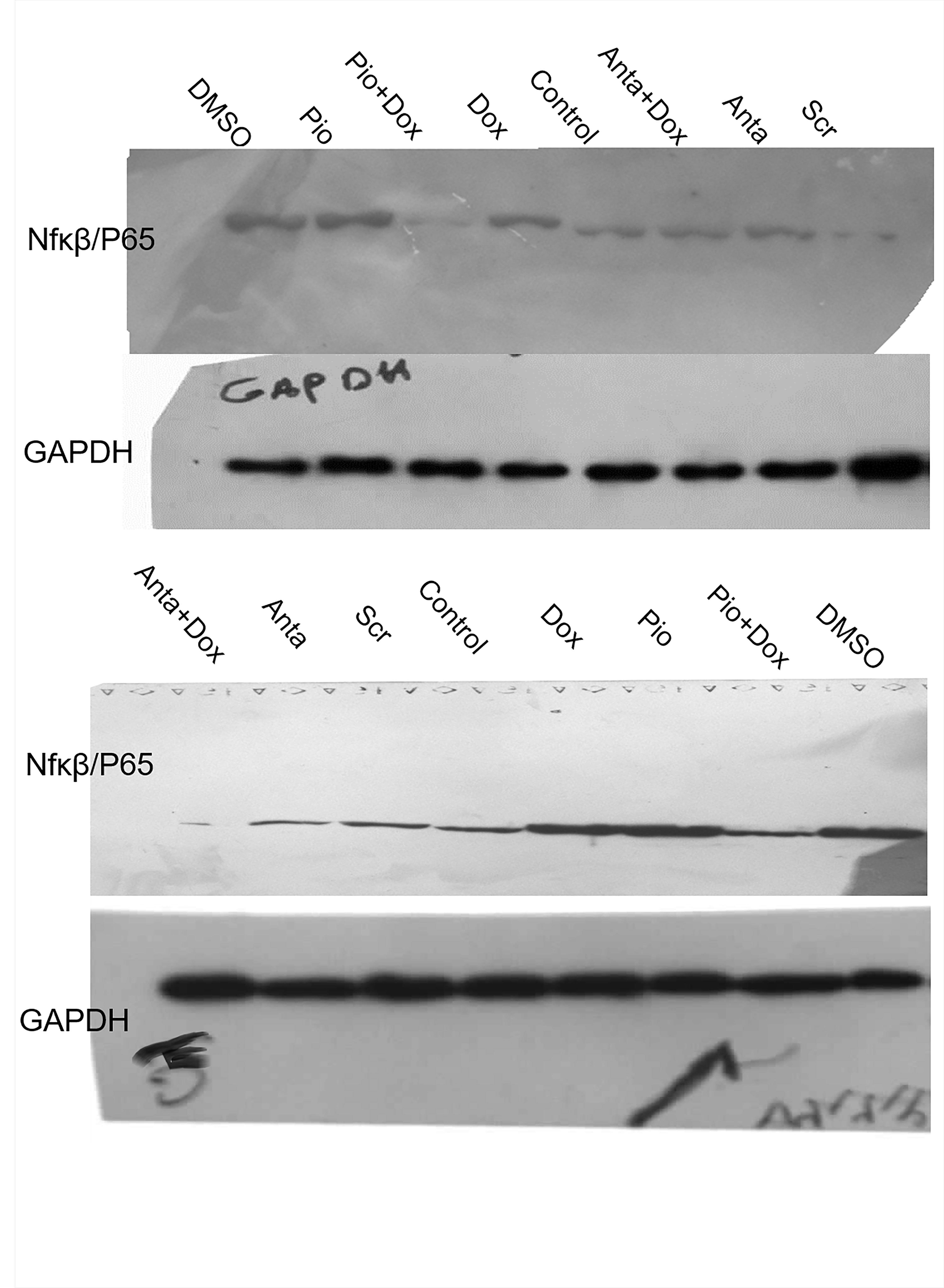

Supplement: Supplementary file 5 — Supplementary Figure 5 [file 41419_2018_797_MOESM5_ESM.tif]
