# Supplementary material for: Downregulation of miR-130a, antagonized doxorubicin-induced cardiotoxicity via increasing the PPARγ expression in mESCs-derived cardiac cells
Source: Cell Death Dis. 2018 Jul 9;9(7):758. doi: 10.1038/s41419-018-0797-1 (PMC6037713; doi:10.1038/s41419-018-0797-1)
Supplement: Supplementary file 6 — Supplementary figure legends [file 41419_2018_797_MOESM6_ESM.doc]

**Supplementary Figure Legends**

**Supplementary Fig. 1.** **Cardiac differentiation of mESCs.**

(A) Schematic representation of the protocol of spontaneous differentiation to beating bodies. (B) Morphologic observation of the cell under light microscope. Cells were visualized on day 2, 12, 15 of the experiment. As evident EBs were emerged on day 2 and gradually cells were enlarged to shape the cardiac cells on day 15. (C) Expression of cardiac markers (*αMHC* and *αcardiac actin*) were significantly detected on days 12 and 15 by RT-qPCR approach. The reference gene was *GAPDH*. Data are represented as mean±SEM of three independent replicates of experiment. Star indicates significant difference of the sample of day 0 with both samples of day 12 and day 15 at *p*<0.05. Scale bar is equal to 200 µm.

**Supplementary Fig. 2. Schematic representation of the protocol used for spontaneous differentiation of mESCs to beating bodies and Dox treatment to obtain the optimal concentration for induction of cardiac toxicity.**

(A) Schematic representation of the protocol of spontaneous differentiation to beating bodies as described in materials and methods. As shown, ascorbic acid (10 µM) used for acceleration of cardiac differentiation for 5 days (day 2 to day 7). On day 14, a limited range of Dox concentrations were implemented to induce cell apoptosis. (B) Within 3 independent replications of this experiment, half maximal inhibitory concentration of Dox was achieved at 5 µM (Boxed column). Hereafter, Dox at 5 µM was used throughout the rest of experiments. (C) Quantification of cell apoptosis, 24 h after Dox treatment (5 µM) by flow cytometry with Annexin V as described in materials and methods. As obvious, Dox treatment enhanced the apoptotic rate of cardiac cells (D) At the same condition as panel C, caspase 3 activity was measured. Of note, there was an increased caspase 3 activity upon Dox induction. (E) RT-qPCR to assess the relative expression of *BCL-2* (anti-apoptotic marker) and *BAX* (pro-apoptotic marker), and *NFκB-P65*, a subunit of NFκB transcription complex 24 h after treatment with Dox. The values are from experiments done in triplicate ± SEM. Reference gene in this study was *GAPDH*. (F) Average relative amount of phosphorylated level of P65, a subunit of NFκB transcription complex to *GAPDH* was estimated in lysate of cardiac cells after Dox treatment compare with the control. The intensity of each band was quantified by Image J software. Remarkably, Dox treatment increased phosphorylated level of P65 thereby, indicted activation of NFκB upon Dox treatment. Data are represented as mean±SEM of three independent replicates of experiment. Star indicates significant difference with control at *p*<0.05.

**Supplementary Fig. 3.** **Determination of optimal concentration of miR-130a-specific antagomir.**

**(**A) Application of three concentrations (5, 10, 25 nM) of miR-130a-specific antagomir for maximal reduction of miR-130a. As indicated, antagomir at two different concentrations (10, 25 nM) was able to reduce the expression of miR-130a in compare with the scramble (Scr) significantly. (B) The same set of experiment was performed to unravel the optimal inducing concentration of anatgomir on *PPARγ* expression in which, antagomir at 25 nM was able to enhance transcript level of *PPARγ* . RT-qPCR was performed thrice independently and control is referred to untransfected cells. Reference genes were U6 and *GAPDH*. Star indicates significant difference with scramble counterpart at *p*<0.05.

**Supplementary Fig. 4. In the normal state, miR-130a-specific antagomir was not able to modify the apoptosis, while moderately reduced effect of inflammatory markers.**

(A) The condition of antagomir application was the same as explained in Fig. 2. As shown in normal condition, antagomir was not able to induce or suppress apoptosis, emphasizing that the concentration (25 nM) used was not toxic for the cell. Apoptosis rate was measured with Annexin V-FITC by flow cytometry. (B) Caspase 3 activity was also measured in transfected cells as described in materials and methods. As evident, no significant changes was observed by transfection with antagomir. (C) RT-qPCR to assess the relative expression of *BCL-2* (anti-apoptotic marker) and *BAX* (pro-apoptotic marker), and *NFκB-P65*, a subunit of NFκB transcription complex 24 h after treatment with Dox. (D) Average relative amount of phosphorylated level of P65, a subunit of NFκB transcription complex to *GAPDH* was estimated in lysate of cardiac cells after antagomir transfection compare with the control. The intensity of each band was quantified by Image J software. Remarkably, antagomir transfection decreased phosphorylated level of P65 thereby, indicted down-regulation of NFκB activity induced by antagomir transfection. Data are represented as mean±SEM of three independent replicates of experiment. Star indicates significant difference with both of scramble and control at *p*<0.05. Reference gene and protein used was GAPDH.

**Supplementary Fig. 5. Original immunoblats of the Western Results**

As indicated at immunoblots of phosphorylated level of P65, a subunit of NFκB transcription complex to GAPDH in lysate of cardiac cells are indicated for two independent replicates for further comparison of the band intensities. These blots were used for fig. 4 and fig. 5 and supplementary fig. 2 and supplementary fig. 4.
