# Supplementary material for: Downregulation of miR-130a, antagonized doxorubicin-induced cardiotoxicity via increasing the PPARγ expression in mESCs-derived cardiac cells
Source: Cell Death Dis. 2018 Jul 9;9(7):758. doi: 10.1038/s41419-018-0797-1 (PMC6037713; doi:10.1038/s41419-018-0797-1)
Supplement: Supplementary file 7 — Supplementary table [file 41419_2018_797_MOESM7_ESM.doc]

**Supplementary table 1.** **Primer sequences**

| **Gene** | **Forward primer** | **Reverse primer** | **Accession number** | |
| --- | --- | --- | --- | --- |
| ***GAPDH*** | **5'-TGCCGCCTGGAGAAACC-3'** | **5'-TGAAGTCGCAGGAGACAACC-3'** | **NM_001289726.1** | |
| ***α-MHC*** | **5'-CAGAAGCCTCGCAATGTC-3'** | **5'-CGGTATCAGCAGAAGCATAG-3'** | **NM_010856.4** | |
| ***α-Actin*** | **5'-GTGTGACGACGAGGAGAC-3'** | **5'-CGATGGACGGGAAGACAG-3'** | **NM_009608.4** | |
| ***BAX*** | **5'-TTTTGCTACAGGGTTTCATC-3'** | **5'-GTCCAGTTCATCTCCAATTC-3'** | **NM_007527.3** | |
| ***P65*** | **5'-GAAGCACAGATACCACCAAGAC-3'** | **5'-TCAGCCTCATAGTAGCCATCC-3'** | **NM_009045.4** | |
| ***BCL2*** | **5'-ACTTCTCTCGTCGCTACCGTC-3'** | **5'-AAGAGTTCCTCCACCACCGT-3'** | | **NM_009741.5** |
